# The British Parliament and the British Army

**Published:** 1897-01-23

**Authors:** 


### The British Parliament and the British Army.

The Army Medical Department report, which has just been issued, is, on the whole, of a satisfactory character. Both in regard to admissions, deaths, and invalidings, the proportion was less than the average for the last ten years. On the other hand, the number constantly non-effective from sickness was greater, as also was the number of days of sickness to each patient and the average duration of each case. Only three cases of small-pox occurred in the United Kingdom, against eleven the year before; but scarlet fever and measles were more prevalent. Continued fever was prevalent at Malta, enteric fever at Bermuda, malarial fevers at Mauritius, and a large increase occurred in the Straits Settlements in the sickness from venereal diseases.

This brings us to the consideration of what, without doubt, is the great blot on the health statistics of the British Army. That the blame does not altogether lie with the soldiers, but depends very largely upon their surroundings and the temptations to which they are exposed, is shown by the extraordinary differences which exist in regard to the proportion affected at different stations. The number of admissions and of those constantly sick from venereal diseases were as follows at the different stations during the year 1895:—

| | Admission per 1,000. | Constantly sick per 1,000. |
|---|---|---|
| England | 164·3 | 14·61 |
| Scotland | 109·7 | 7·86 |
| Ireland | 126·8 | 10·98 |
| Gibraltar | 180·6 | 16·49 |
| Malta | 126·4 | 12·55 |
| Malta Royal Artillery stationed at Malta | 34·6 | 2·10 |
| Canada | 124·7 | 5·8 |
| Bermuda | 60·1 | 5·46 |
| West Indies | 245·5 | 18·42 |
| Non-European troops, West Indies | 307·7 | 24·63 |
| ,, ,, West Africa | 188·6 | 18·48 |
| South Africa and St. Helena | 279·7 | 27·41 |
| Mauritius | 173·5 | 15·89 |
| Ceylon | 249·6 | 21·57 |
| China | 300·3 | 23·08 |
| Straits Settlements | 589·9 | 43·39 |
| India | 470·0 | 40·83 |

This shows the frightful bill which England has to pay for the temptations which she allows to be placed in the way of her young soldiers. In India nearly half, and in the Straits Settlements far more than half, of the entire force suffered from venereal disease during the year. Nowhere do English soldiers cost more than in India, and nowhere are they more carefully and anxiously protected from every disease except this. Yet we stand by with our hands folded while in India, where more than anywhere, we want our soldiers to be always ready, upwards of three battalions (2,789 men) are constantly lying incapacitated from venereal disease. To show again how much depends on the surroundings and temptations amid which we allow our soldiers to live rather than on innate vice on their part, it should be noted that even in India the number admitted per 1,000 varied in different places from 286 to 715! Surely it is time that something was done to stop this disgraceful state of affairs.

